# The Technique of Amputations, Particularly the Treatment of Nerves

**Published:** 1918

**Authors:** 


***The Technique of Amputations, Particularly the Treatment of Nerves.*** By Edred M. Corner. Abs. from the *Journal R. A. M. C.*, May, 1918.

The author emphasizes the importance of early closing of nerve ends as a means of avoiding subsequent nerve irritation resulting from amputation. He argues that a nerve end left open in a healing and especially a suppurating wound treated with chemical irrigation, even if the nerve is cut short, receives a certain amount of irritation which may lead to serious and lasting consequences. Sometimes such irritation may respond to surgical and medical treatment, but often it is beyond the reach of the surgeon.

He believes that the logical procedure is to close the open nerve, as by a ligature after proximal and several crushings of the nerve with artery forceps. As the divided end of the nerve will be sealed in a few hours, the ligature should be of the finest and most quickly absorbed catgut.

The end of the nerve, capped by sterile crushed nerve tissue, is then covered, sealed, and held in place by the ligatured nerve sheath. It is best to block the large nerve trunks with novocain injections before crushing them.

Instead, if the nerve end is large enough for manipulation, nerve flaps may be cut so as to close the open end. The nerve is divided by two cuts, making two rather long flaps which are brought together, closing the nerve, as it retracts, by the pressure of the surrounding structures. Flaps made in the perineurium cannot be relied upon to close the nerve.

*Bone closing* has also been unsatisfactory. Periosteum and osteoplastic flaps have never succeeded. The author believes that the saw used in amputations has been responsible for the thrombosis of vessels by the heat generated by the friction, and hence the necrosis. He prefers the guillotine technique.

Divided muscle, left open, is subject to the same dangers as open nerves and bone. He believes that cut muscle should be sealed with a continuous catgut suture. Such a procedure greatly lessens the amount of fluid exuded and makes the identification of the divided structures easier.

In conclusion the author states that the skin of no amputation wound should be sutured until all open structures, except, perhaps, bone, have been sealed.

